# Data on growth performance, proximate composition, and fatty acid content of edible oyster (*Crassostrea* spp.), farmed on shellstring along Cox's Bazar Coast

**DOI:** 10.1016/j.dib.2020.106450

**Published:** 2020-10-26

**Authors:** Tashrif Mahmud Minhaz, Joyshri Sarker, Mohammed Nurul Absar Khan, Helena Khatoon, Md Abdul Alim, SM. Khalequzzaman, Moin Uddin Ahmad, Mohammad Redwanur Rahman

**Affiliations:** a Department of Aquaculture, Faculty of Fisheries, Chattogram Veterinary and Animal Sciences University, Bangladesh; b Department of Fishing & Post-Harvest Technology, Faculty of Fisheries, Chattogram Veterinary and Animal Sciences University, Bangladesh; c Department of Fisheries, Ministry of Fisheries and Livestock, Bangladesh

**Keywords:** Shellstring method, *Crassostrea* spp., Growth performance, Proximate composition, Fatty acid, Cox's Bazar coast

## Abstract

Data on growth performance, proximate composition and fatty acid content of Edible oysters (*Crassostrea* spp.) were collected to evaluate the spatial variation in growth performance, proximate composition and fatty acid content of oyster. The oyster was farmed on shellstring for 120 days in the three sites: Nunia chara, Chowfoldandy and Sonadia Island. Oysters were marked into six different age classes: T_1_: 31—45 days; T_2_: 46—60 days; T_3_: 61—75 days; T_4_: 76—90 days; T_5_: 91—105 days and T_6_: 106—120 days. Data on environmental variables were collected in every 15 days. Oysters were collected for physical measurements and biochemical analysis after 120 days. Data on growth performance showed spatial variation. Withal, data on proximate composition and fatty acid content were significantly different (p < 0.05) among the three sites. This data could contribute in oyster aquaculture development.

## Specifications Table

**Table:** 

Subject	Food Science, Aquatic Science
More specific subject area	Oyster growth, proximate and fatty acid
Type of data	Table, Chart and Image
How data were acquired	Physical measurements and biochemical analysis for environmental variables, physical measurements as well as calculation for growth performance, biochemical analysis for proximate composition and gas chromatographic mass spectrophotometric (GCMS) analysis for fatty acid profile.
Data format	Raw (individual measurements) and analyzed
Parameters for data collection	Shellstring arrays were deployed at three different sites in Cox's Bazar coast with three replicates in each site for 120 days. Oysters were grouped into six age classes (T_1_—T_6_). 5% oysters of all age classes were collected from all the replicates for growth parameters and biochemical analysis.
Description of data collection	For growth performance: total weight gain, dry meat mass gain, length increment, width increment and thickness increment. For proximate composition: moisture, protein, lipid, ash, fiber and carbohydrate. For fatty acid: GCMS analysis on lipid extracted from oyster
Data source location	Nunia Chara (NC – 21°28ˈ19.5" N, 91°57ˈ42.7" E); Chowfoldandy (CD – 21°30ˈ44.1" N, 92°01ˈ00.1" E); Sonadia Island (SI – 21°30ˈ18.7" N, 91°53ˈ43.3" E) at Cox's Bazar coast, Bangladesh
Data accessibility	Available with this article and also at https://data.mendeley.com/drafts/kdwgk8rh7f

## Value of the Data

• Growth performance of oyster (*Crassostrea* spp.), presented in this data, could be useful to estimate possible culture period for commercial oyster farming in Bangladesh.
• Understanding of variation in proximate composition as well as fatty acid of oyster from different culture sites in this data. This data could be valuable in assessing health benefits of oyster, farmed in Cox's Bazar, Bangladesh.
• Spatial variation in growth performance, proximate composition and fatty acid of oyster in this data, could be useful to allocate commercial culture sites.

## 1 Data Description

Environmental condition of this data collection sites was shown in Table 1. Variation in salinity (3–35 g/L), temperature (21–32.5 °C), pH (6.4–8.2), high tide water depth (91.4–642.6 cm), low tide water depth (5.1–360.7 cm), total suspended solids (33.7–171 g/L), chlorophyll a (0.42–7.64 μg/L), nitrate nitrogen (0.009–0.514 ppm) and soluble reactive phosphorus (0.099– 0.876 ppm) were observed across the three sites.

**Table 1:** Environmental condition at NC, CD and SI site from 24^th^ September 2019 to 23^rd^ January 2020.

		Environmental variables								
Data collection sites	Level of values	Sal	Temp	pH	HTWD	LTWD	TSS	Chl a	NO_2_-N	SRP
NC	Max	35.0	32.0	8.2	302.3	45.7	171.0	5.93	0.381	0.183
	Average	27.8 ± 2.1	27.3 ± 1.4	7.7 ± 0.1	205.5 ± 23.9	15.8 ± 5.5	73.7 ± 13.5	3.03 ± 0.62	0.142 ± 0.078	0.149 ± 0.018
	Min	15.0	22.0	7.2	91.4	5.1	33.7	0.42	0.009	0.099
CD	Max	32.0	32.5	7.9	642.6	360.7	162.7	7.64	0.514	0.876
	Average	20.3 ± 3.3	26.9 ± 1.6	7.4 ± 0.1	589.8 ± 16.3	296.6 ± 13.3	79.6 ± 12.0	3.53 ± 0.76	0.229 ± 0.099	0.435 ± 0.112
	Min	3.0	21.0	6.4	520.7	248.9	42.7	0.88	0.060	0.107
SI	Max	34.0	33.0	8.0	184.0	66.0	142.0	6.02	0.430	0.920
	Average	25.5 ± 2.3	27.4 ± 1.5	7.7 ± 0.1	141.2 ± 10.1	48.2 ± 3.4	64.6 ± 10.6	2.78 ± 0.63	0.199 ± 0.085	0.260 ± 0.149
	Min	14.0	22.0	7.4	92.0	32.0	37.7	0.45	0.028	0.103

Sal: Salinity in g/L; Temp: Temperature in°C; HTWD: High tide water depth in cm; LTWD: Low tide water depth in cm; TSS: Total suspended solids in g/L; Chl a: Chlorophyll a in μg/L; NO_2_–N: Nitrite nitrogen in mg/L; SRP: Soluble reactive phosphorus in mg/L.

**Fig. 1:**
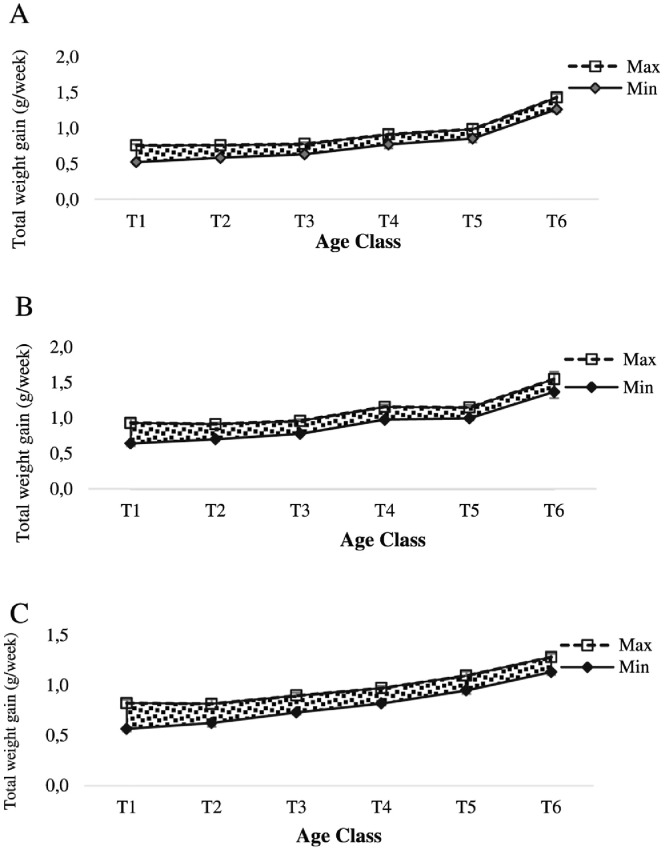
Range of total weight gain of oyster at Nunia Chara (A), Chowfoldandy (B) and Sonadia Island (C) sites.

**Fig. 2:**
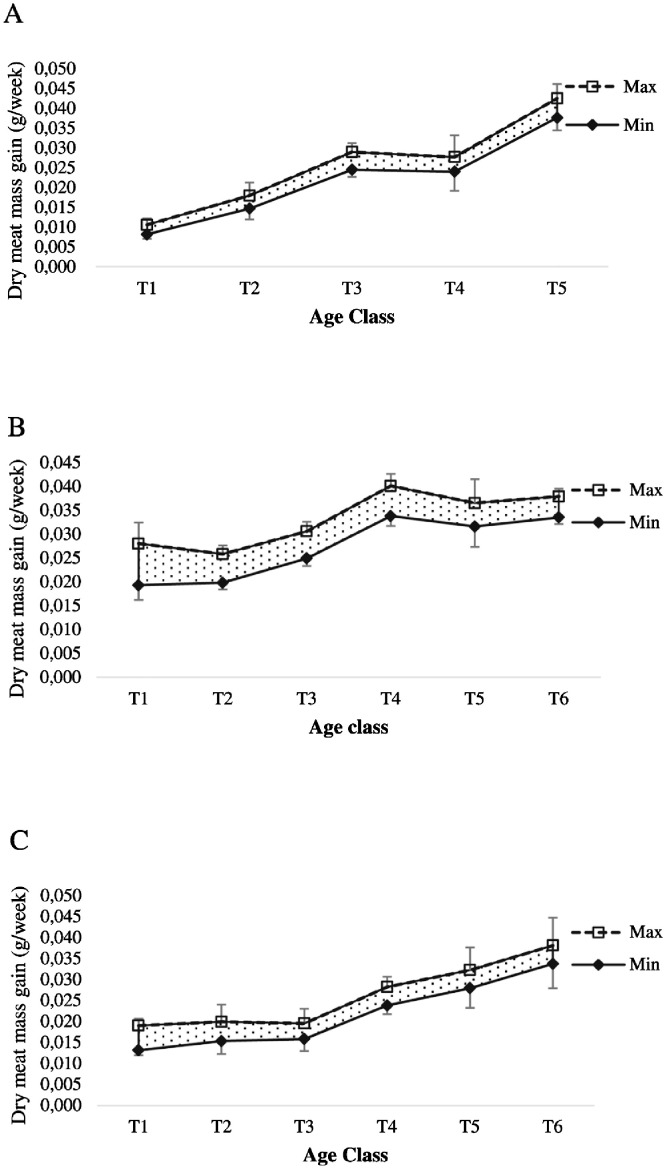
Range of dry meat mass gain of oyster at Nunia Chara (A), Chowfoldandy (B) and Sonadia Island (C) sites.

**Fig. 3:**
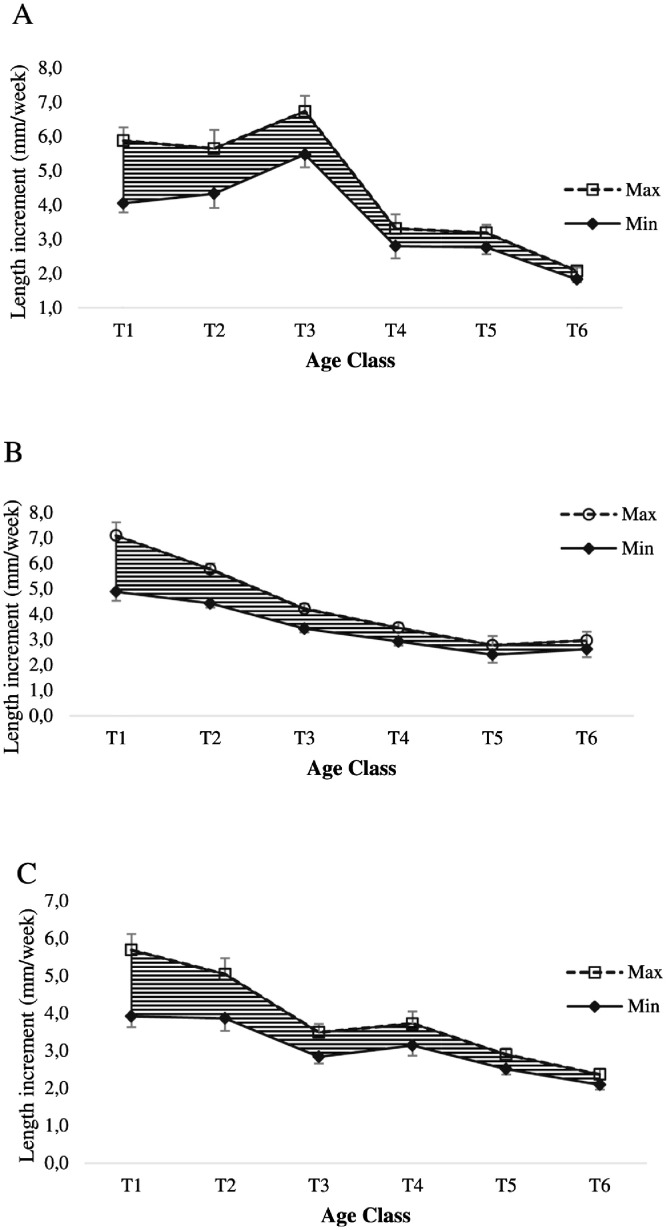
Range of length increment of oyster at Nunia Chara (A), Chowfoldandy (B) and Sonadia Island (C) sites.

**Fig. 4:**
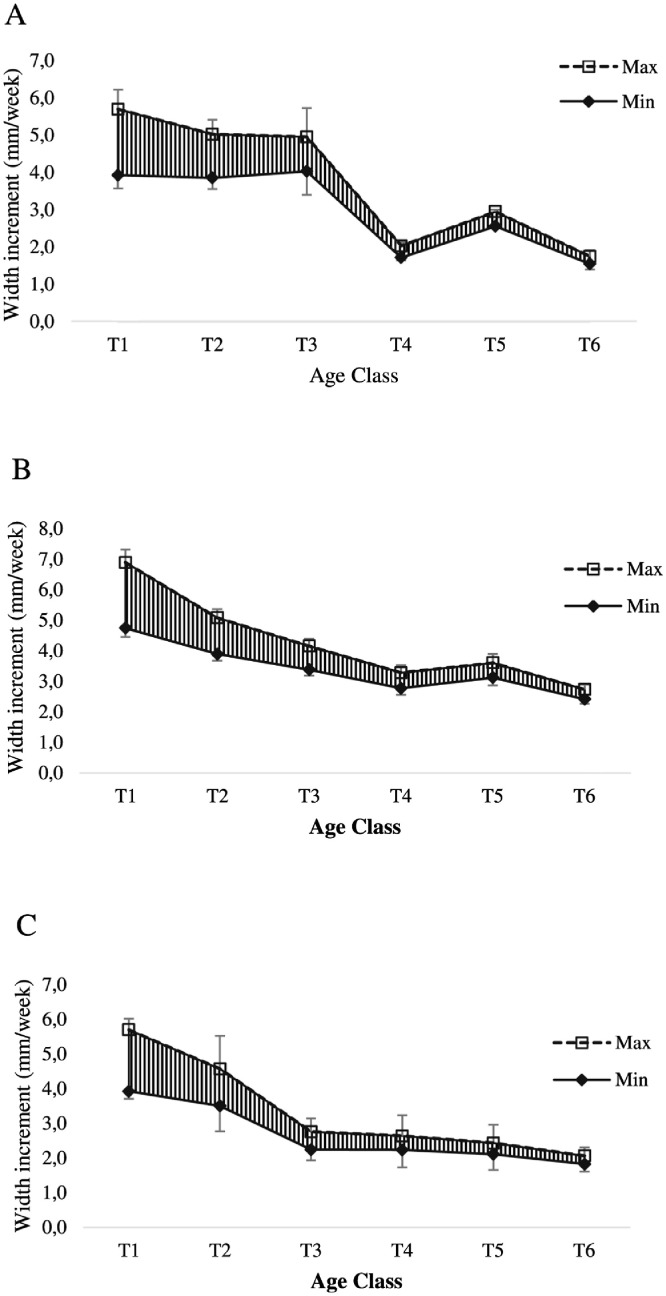
Range of width increment of oyster at Nunia Chara (A), Chowfoldandy (B) and Sonadia Island (C) sites.

**Fig. 5:**
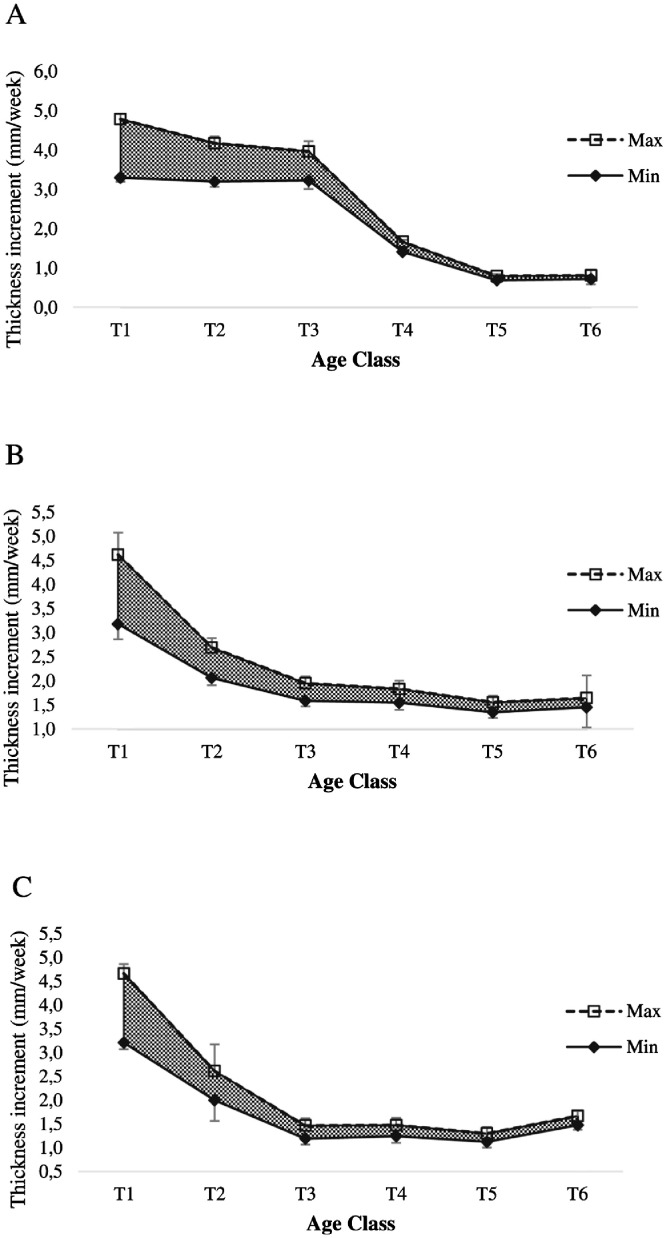
Range of thickness increment of oyster at Nunia Chara (A), Chowfoldandy (B) and Sonadia Island (C) sites.

Growth performance of oyster were defined by total weight gain (g/week), dry meat mass gain (g/week), length increment (mm/week), width increment (mm/week) and thickness increment (mm/week). This data shows maximum and minimum value range for all these parameters (Fig. 1-5).

**Fig. 6:**
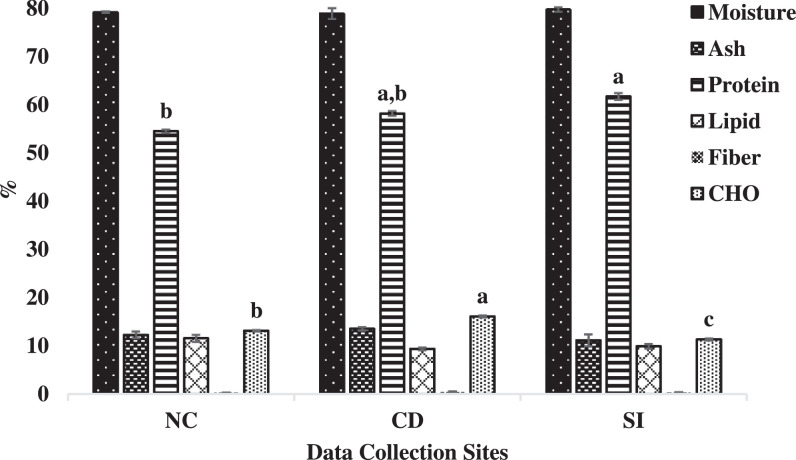
Proximate content of oyster at the three sites (NC–Nunia Chara, CD– Chowfoldandy and SI– Sonadia Island). Moisture content was represented in wet weight basis and rests were represented in dry weight basis. Values are means of three replicates with error bar (standard error; SE = σ/√n). Values with different letters within each series are significantly different (p < 0.05).

This data shows moisture, protein, lipid, carbohydrate, ash and fiber content of oyster from the three sites. Moisture (78.8–79.6%, wet weight basis), ash (11.1–13.5%, dry weight basis), lipid (9.3–11.5%, dry weight basis) and fiber content (0.3–0.4%, dry weight basis) of oyster were not significantly (p < 0.05) different among the three sites. Protein and carbohydrate content were significantly (p < 0.05) different among the three sites. Highest protein (61.6 ± 0.7%, dry weight basis) and carbohydrate content (16.1 ± 0.2, dry weight basis) was found in SI and CD sites respectively, while lowest protein (54.4 ± 0.3%, dry weight basis) and carbohydrate content (11.3 ± 0.2, dry weight basis) was found in NC and SI sites respectively (Fig. 6).

**Fig. 7:**
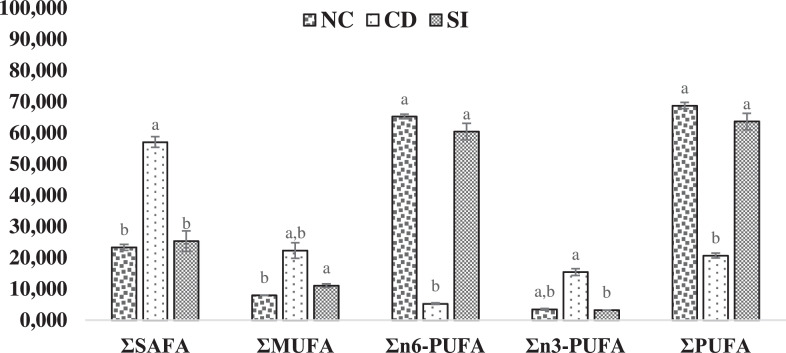
Fatty acid content (% of total fatty acids) in oyster from the site NC– Nunia Chara, CD– Chowfoldandy and SI– Sonadia Island. Values are means of duplicates with error bar (standard error; SE = σ/√n). Values with different letters within each category are significantly different (p < 0.05). SAFA– Saturated Fatty Acids, MUFA– Mono Unsaturated Fatty Acids, PUFA- Poly Unsaturated Fatty Acids.

**Fig. 8:**
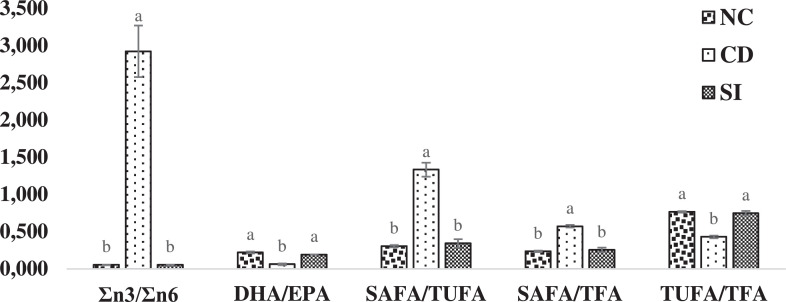
Fatty acid ratios in oyster from the site NC– Nunia Chara, CD– Chowfoldandy and SI– Sonadia Island. Values are means of duplicates with error bar (standard error; SE = σ/√n). Values with different letters within each category are significantly different (p < 0.05). SAFA– Saturated Fatty Acids, DHA– Docosahexaenoic Acid, EPA– Eicosapentaenoic Acid, TUFA– Total Unsaturated Fatty Acids, TFA– Total Fatty Acids, n3– Omega 3 fatty acids, n6– Omega 6 fatty acids.

Finally, this data shows fatty acid content of oyster from the three sites, which were analyzed by GCMS (Table 2, Fig. 7–8).

**Table 2:** Fatty acid content of oyster (% of total fatty acids) from the three sites.

Carbon	FA	NC	CD	SI
C8:0	Octanoic acid	1.14 ± 0.04	1.30 ± 0.09	1.08 ± 0.03
C10:0	Decanoic acid	1.01 ± 0.03	1.16 ± 0.08	1.02 ± 0.02
C12:0	Lauric acid	3.63 ± 0.06	5.89 ± 0.43	3.83 ± 0.10
C13:0	Tridecanoic acid	0.66 ± 0.03	1.00 ± 0.06	1.23 ± 0.03
C14:0	Myristic acid	7.69 ± 0.16	20.90 ± 1.53	8.31 ± 2.22
C16:0	Palmitic acid	4.21 ± 0.08	13.23 ± 5.03	5.36 ± 1.41
C18:0	Stearic acid	0.83 ± 0.01	2.97 ± 0.18	0.46 ± 0.08
C20:0	Arachidic acid	1.90 ± 0.02	1.73 ± 0.20	1.33 ± 0.07
C17:0	Heptadecanoic acid	0.02 ± 0.00	3.63 ± 0.26	0.01 ± 0.00
C21:0	Heneicosanoic acid	0.03 ± 0.03	0.02 ± 0.00	0.06 ± 0.00
C22:0	Behenic acid	0.85 ± 0.01	2.62 ± 0.16	1.04 ± 0.03
C23:0	Tricosanoic acid	0.36 ± 0.02	0.47 ± 0.02	0.41 ± 0.00
C24:0	Lignoceric acid	1.01 ± 0.48	2.09 ± 0.33	1.18 ± 0.03
C16:1	Palmitoleic acid	0.98 ± 0.01	15.88 ± 1.17	7.24 ± 0.35
C18:1	Oleic acid	0.69 ± 0.03	0.75 ± 0.04	0.39 ± 0.11
C20:1	cis-11-Eicosenoic acid	4.72 ± 0.37	3.54 ± 0.06	1.80 ± 0.13
C22:1	Erucic acid	1.47 ± 0.39	1.43 ± 0.59	1.40 ± 0.51
C24:1	Nervonic acid	0.10 ± 0.01	0.70 ± 0.65	0.22 ± 0.20
C18:2n-6	Linoleic acid	62.66 ± 0.83	0.32 ± 0.02	57.85 ± 2.60
C20:3n-6	Eicosatrienoic acid	0.54 ± 0.07	1.53 ± 0.13	0.78 ± 0.02
C20:4n-6	Arachidonic acid	1.98 ± 0.17	3.45 ± 0.11	1.75 ± 0.00
C18:3n-3	Linolenic acid	0.41 ± 0.01	2.67 ± 0.48	0.45 ± 0.21
C20:5n-3	Eicosapentanoic acid	2.10 ± 0.09	11.06 ± 0.73	2.29 ± 0.19
C22:5n-3	Docosapentaenoic acid	0.57 ± 0.17	0.98 ± 0.09	0.08 ± 0.05
C22:6n-3	Docosahexaenoic acid	0.46 ± 0.00	0.68 ± 0.09	0.44 ± 0.04

Values are means of duplicates with error bar (standard error; SE = σ/√n). (NC–Nunia Chara, CD– Chowfoldandy and SI– Sonadia Island).

## 2 Materials and methods

### 2.1 Data collection sites

**Fig. 9:**
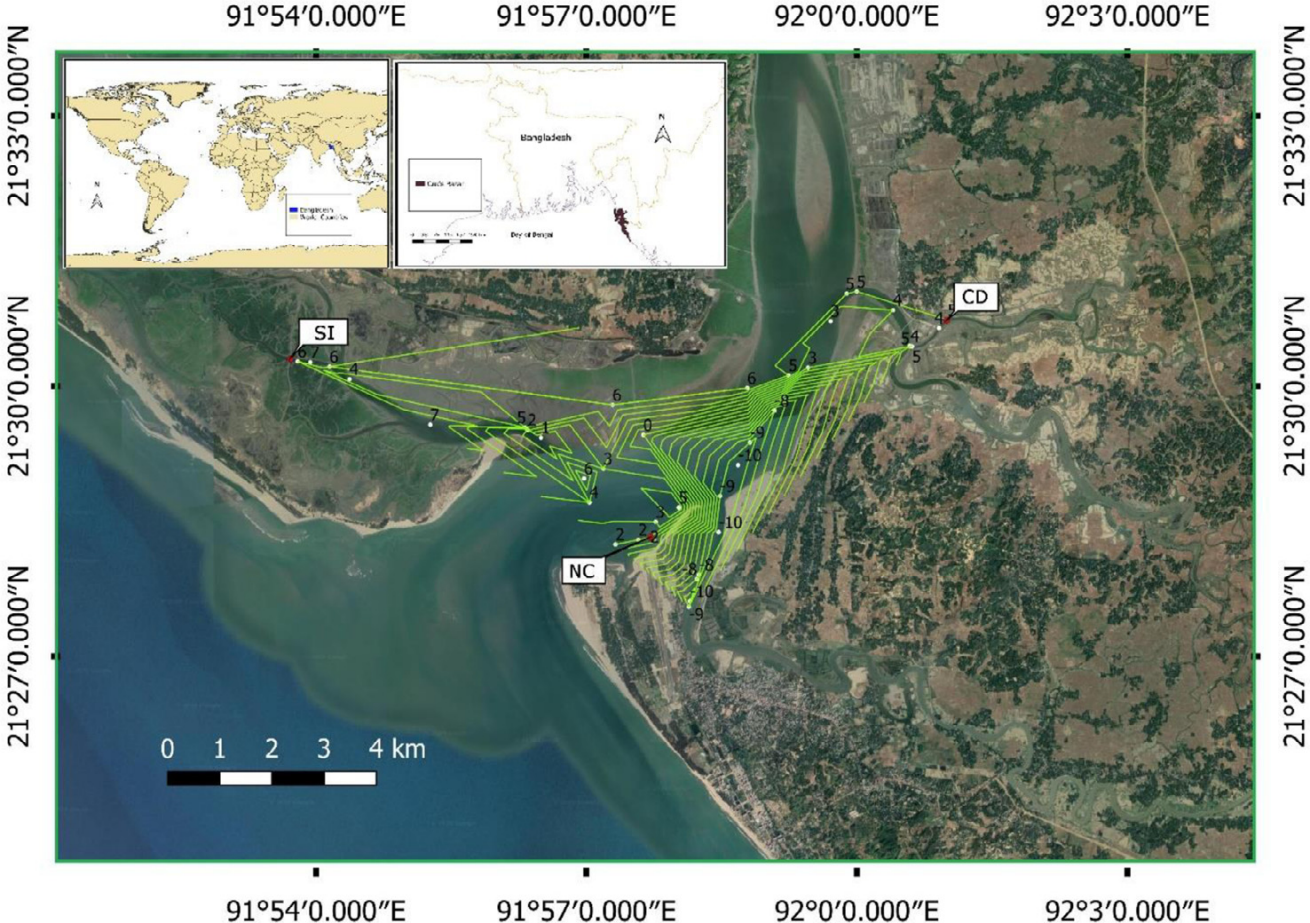
Map of the data collection sites. NC, CD and SI represents Nunia Chara, Chowfoldandy, and Sonadia Island sites respectively.

Cox's Bazar coast is prevailed by a subtropical monsoonal climate. From December to February, the climate is mild and dry, characterized by minimum air temperatures from 10 °C to 16.4 °C, and during summer, maximum air temperature reaches 38.5 °C. In early June, heavy southwest monsoon rains begin and continue to mid–October. During the monsoon months (i.e., June–September), 80% of the total rainfall occurs with the annual rainfall varies between 2320 and 5447 mm [1]. Typically, a semi–diurnal tide pattern is observed in these coastal waters. Seasonal variations in mean tide level is 50–80 cm with approximately 3.5 m tidal range [2]. Three different sites: (a) Nunia Chara (NC–21°28ˈ19.5" N, 91°57ˈ42.7" E); (b) Chowfoldandy (CD–21°30ˈ44.1" N, 92°01ˈ00.1" E): (c) Sonadia Island (SI–21°30ˈ18.7" N, 91°53ˈ43.3" E) were chosen to establish substrate units (see Fig. 9). NC is an intertidal zone characterized by a muddy bottom and becomes dry during low tide throughout the neap tide. It is moderately influenced by surface runoff carried through the Maheshkhali channel. CD is a subtidal zone characterized by a rocky and muddy bottom. It is strongly influenced by surface runoff. SI is also a subtidal zone characterized by a muddy bottom and surrounded by mangroves. It is slightly influenced by surface runoff. Random dispersion of *Crassostrea* spp. is observed in NC, whereas clumped dispersion is observed in CD and SI sites.

### 2.2 Experimental design

**Fig. 10:**
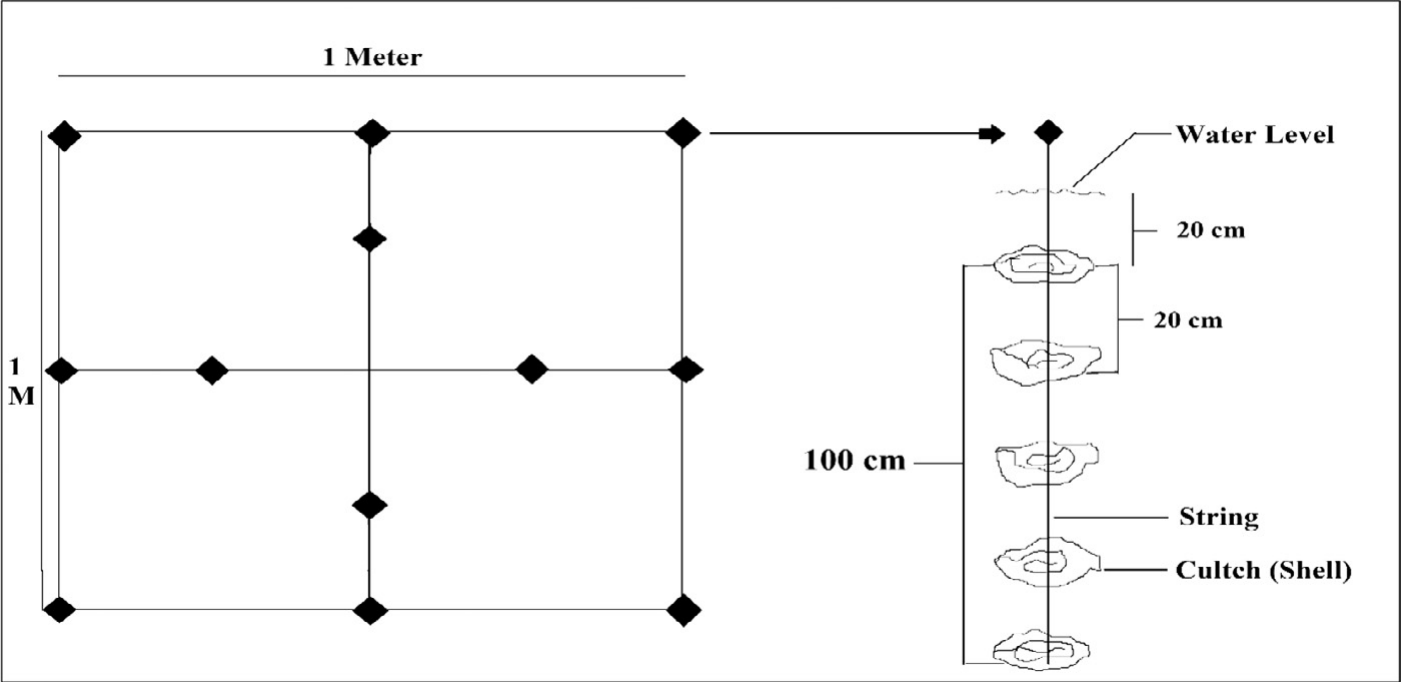
Upper (left side) and side view (right side) of a substrate unit holding 12 shellstrings and hanged from a bamboo raft.

Triplicates of substrate unit were used in all the three sites maintaining 1–meter distance between two units. Shellstring arrays were deployed (modified from [3]) in each substrate unit that contained 12 strings placed in a pattern as showed in Fig. 10. The modification was made in the number of shell per string. Each string contained 5 oyster shells at 20 cm distance from each other, and the first one was placed at 20 cm water depth from the surface. Thus, each substrate unit consisted of 60 oyster shells. Each of the strings was tagged with a unique identification. Each shell surface area was measured using the Aluminum Foil Mold method [4] and the sum of the surface area of both sides of 60 shells was the total surface area of a substrate unit. Mean shell surface area of three substrate units at NC, CD, and SI sites were 5889.9 ± 265.9 cm^2^, 4865.0 ± 100.6 cm^2^ and 5095.5 ± 357.2 cm^2^ respectively. The floating bamboo raft was used to set the substrate unit and was anchored to the bottom mud in such a way that it could easily move up and down along with tidal fluctuation. Oysters spat that were recruited after 24^th^ September 2019 were tagged into six different age class with 15 days interval using permanent marker pen of different colors. Where, T_1_: 31—45 days (marked on 22^nd^ December 2019); T_2_: 46—60 days (marked on 7^th^ December 2019); T_3_: 61—75 days (marked on 23^rd^ November 2019); T_4_: 76—90 days (marked on 8^th^ November 2019); T_5_: 91—105 days (marked on 23^rd^ October 2019) and T_6_: 106—120 days (marked on 8^th^ October 2019). During tagging, total number of spat settled on shell was counted. Number of dead spats was subtracted from that data during the following tagging or sampling.

### 2.3 Determination of environmental variables

High and low tide water depth, water salinity, temperature and pH were measured in situ during every sampling. During the water depth measurement, either high tide or low tide water depth was measured manually. The tidal range of sampling day was taken from the real-time tide chart available at https://www.tide-forecast.com and then either added with low tide water depth to get high tide water depth or subtracted from high tide water depth to get low tide water depth. The water temperature, water pH and water salinity was measured from surface water by using a glass thermometer, a handheld pH meter (pHep-HI98107, HANNA) and a handheld ATC refractometer (YEGREN), respectively. Chlorophyll a was determined by 90% acetone method (modified from [5]). The modification was made in the amount of filtered water from sample. 500 ml sample water was filtered instead of 1000 ml. NO_2_-N and soluble reactive phosphorous (SRP) was determined according to Parsons et al. (1984). All the instruments used were calibrated before using.

### 2.4 Oyster collection

Oysters were collected on 23^rd^ January 20209 (after 120 days from the deployment) from shellstring and stored in ice for growth parameter. 5% samples were collected from all age classes from 3 replicated experimental units in all the three sites. This was calculated from the data of total live spat count in each age class which was maintained during each sampling period. Oyster of T_5_ age class from all the three sites were taken for proximate and fatty acid analysis.

### 2.5 Growth parameters

Data of oyster total weight, dry meat mass, length, width and thickness were measured using electric balance and digital slide calipers in the laboratory. Then, on shell total weight gain, dry meat mass gain, length increment, width increment and thickness increment were calculated. Following formula [6] were used for calculation:

Total weight gain (g/week) = (Total body weight/Minimum or maximum age) × 7
Dry meat mass gain (g/week) = (Dry meat mass/Minimum or maximum age) × 7
Length increment (mm/week) = (Total length/Minimum or maximum age) × 7
Width increment (mm/week) = (Total width/Minimum or maximum age) × 7
Thickness increment (mm/week) = (Total thickness/Minimum or maximum age) × 7

### 2.6 Proximate

Oyster samples (whole body) for protein, lipid, and carbohydrate were freeze dried firstly. All the samples were blended into fine powder. Moisture, protein, lipid, ash and crude fiber were determined according to the standard methods of AOAC [7]. Wet oyster samples were dried at 105 °C temperature in hot air oven until reaching to a constant weight. Protein content of dry oyster sample was determined by Kjeldahl method (N x 6.25) using Kjeldahl apparatus and manual titration. Soxhlet apparatus was used to determine lipid at 100 °C and using diethyl ether as solvent. Ash content was determined by using muffle furnace at 550 °C temperature for 6 h. Crude fiber was determined by using fiber extraction apparatus and muffle furnace. Samples were first acid boiled and then alkali boiled at 100 °C and then filtered with acetone. Then the residue was ignited at 600 °C for 3 h in muffle furnace. Carbohydrate analysis was conducted based on the method [8]. For each sample, 5 mg freeze dried powder was taken and made into 25 ml solution by mixing with distilled water. Tissue homogenizer was used for homogenous mixing. Prior to analysis, 5% phenol solution and concentrated sulphuric acid was prepared. Samples were analyzed by adding 1 of 5 % phenolic solution and 5 mL of concentrated sulphuric acid. The standard was prepared using glucose. The optical density was measured at 488 nm using a spectrophotometer (UV-VIS Double beam, Model-T80, HANNA).

### 2.7 Fatty acids

Oyster samples (whole body) of T_5_ age class were freeze dried and blended into fine powder prior to the start of GCMS analysis. Fatty acids were determined according to Prato et al. [9]. At first, lipid was extracted from the sample using Soxhlet apparatus. Diethyl ether was used as solvent during lipid extraction. At the final stage of lipid extraction 60 °C temperature was maintained. This lipid sample was used to analyze fatty acid methyl esters. Analysis of Fatty acids methyl esters (FAMEs) were conducted by gas chromatography mass spectrophotometry using a GCMS-QP2020 (Shimadzu, Japan), equipped with flame ionization detector. FAMEs were separated with a capillary column (Length 30 m, internal diameter 0.25 mm, film thickness 0.15 μm, phase ratio 250). Helium was used as carrier gas at a flow rate of 1.42 ml/min. The column temperature program was as follows: 180 to 280 °C at 5 °C /min and then held at 280 °C. FAMEs were identified by comparing retention times with a standard (FAME mix C8-C24; Sigma-Aldrich, Germany). Quantities were expressed in ppm. Then it was converted into % of total fatty acids.

### 2.8 Statistical analysis

MS Excel was used to calculate the mean, standard error of mean (SE = σ/√n) of the data and homogeneity of variance was assayed by means of Levene's test. One way multivariate analysis of variance was computed to examine proximate and fatty acids to verify whether there were differences among the three sites. The multiple range tests (Tukey's test) were applied when the variance analysis indicated significant differences. The level of significance was set as 0.05. Statistical analyses were accomplished using the SPSS (IBM v. 25.0) statistical software.

## Ethics Statement

These data were collected complying ARRIVE guidelines. As oyster is not protected by any regulation or law in Bangladesh, we didn't take ethical approval from law implementing authority prior to the start of the data collection procedure.

## Declaration of Competing Interest

None.

## Appendix Supplementary materials

Image, application 1
