# Supplementary material for: Data on growth performance, proximate composition, and fatty acid content of edible oyster (Crassostrea spp.), farmed on shellstring along Cox's Bazar Coast
Source: Data Brief. 2020 Oct 26;33:106450. doi: 10.1016/j.dib.2020.106450 (PMC7642811; doi:10.1016/j.dib.2020.106450)
Supplement: Supplementary file 1 [file mmc1.pdf]

DIB-D-20-01752-Data on growth performance, proximate composition, and fatty acid content of edible oyster (Crassostrea spp.), farmed on shellstring along Cox's Bazar Coast

Published: 17-10-2020 | **Version 1** | **DOI:** 10.17632/kdwgk8rh7f.1
Contributors: Tashrif Mahmud Minhaz, Joyshri Sarker, Mohammed Nurul Absar Khan, Helena Khatoon, Md Abdul Alim, SM. Khalequzzaman, Moin Uddin Ahmad, Mohammad Redwanur Rahman

Description
